# Dual Process Theory: Embodied and Predictive; Symbolic and Classical

**DOI:** 10.3389/fpsyg.2022.805386

**Published:** 2022-03-21

**Authors:** Samuel C. Bellini-Leite

**Affiliations:** Utilization Systems Department, Minas Gerais State University, Belo Horizonte, Brazil

**Keywords:** dual process theory, embodied cognition, bounded rationality, predictive processing, cognitive science

## Abstract

Dual Process Theory is currently a popular theory for explaining why we show bounded rationality in reasoning and decision-making tasks. This theory proposes there must be a sharp distinction in thinking to explain two clusters of correlational features. One cluster describes a fast and intuitive process (Type 1), while the other describes a slow and reflective one (Type 2). A problem for this theory is identifying a common principle that binds these features together, explaining why they form a unity, the unity problem. To solve it, a hypothesis is developed combining embodied predictive processing with symbolic classical approaches. The hypothesis, simplified, states that Type 1 processes are bound together because they rely on embodied predictive processing whereas Type 2 processes form a unity because they are accomplished by symbolic classical cognition. To show that this is likely the case, the features of Dual Process Theory are discussed in relation to these frameworks.

## Introduction

Embodied cognition has been proposed as an alternative to symbolic processing since it started to grow in the 90s. Although it is true that embodied cognition contrasts with traditional cognitive science, the possibility that these frameworks might explain different kinds of processes in cognition is overlooked. In the same sense that different framework in physics such as quantum mechanics, general relativity and even the traditional classical mechanics co-exist, each explaining parts of our world, it is likely that 4E cognition, traditional cognitive science, connectionism, and predictive processing can co-exist if we understand to which domains of cognition these apply (Bellini-Leite, 2017). A theory of everything in cognition should most likely attempt to unify parts of these proposals rather than to keep only one.

Evidence in the reasoning and rationality literature has consistently pointed to the idea that human rationality is bounded by proximal stimuli and cognitive limitations. This has led to the interpretation that humans do not have a perfect logical or probabilistic problem-solving system but rather diverse heuristics, algorithms or simple mechanisms that are used to deal with environmental challenges. These conclusions come from experiments which show how people respond in puzzling ways to certain questions. But the reason certain systems are bounded and how they are bounded should vary greatly depending on which systems these are. Thus, we need to consider divisions in cognition as well to understand bounded rationality (Bellini-Leite and Frankish, 2020).

One currently popular way to divide types of cognitive processes is Dual Process Theory (DPT). This theory, proposing there are two distinct processes, Type 1 (T1) and Type 2 (T2), underlying higher-order thinking has recently received much attention for explaining the evidence in reasoning, judgment and decision-making tasks. DPT claims there must be a sharp distinction between two clusters of correlational features. One cluster describes a fast and intuitive process, while the other describes a slow and reflective one (Evans, 2008; Kahneman, 2011; Evans and Stanovich, 2013). Some T2 core features are heavy working memory load, explicitness, low capacity, high effort and slowness, while T1 central features are weak loading on working memory, implicitness, high capacity, low effort, and speed.

However, Samuels (2009) notes that even if one considers the evidence to be convincing and the dichotomy of processes T1 and T2, along with their property clusters (termed S1 and S2), well placed, we still have a basic research question open, which he calls the unity problem:

“though positing mechanisms is a standard strategy for explaining the existence of property clusters, it does not, by itself, constitute a satisfactory explanation. Rather one needs to specify those features of the proposed mechanisms that account for such clustering effects. In the present case, we need to specify those characteristics of type-1 systems that yield S1-exhibiting processes, and those properties of type-2 systems that yield S2-exhibit-ing processes. Again, this does not strike me as a serious objection so much as a challenge for future research—one that requires a more detailed account of the systems responsible for type-1 and type-2 processes.” (Samuels, 2009, p. 141).

The unity problem should not be confused with the reference problem (Samuels, 2009). The reference problem of DPT is the problem of determining what the theory is about, to which a possible answer would be “about distinct systems” or “different minds” or “modes” (see Bellini-Leite, 2018). After answering the reference problem, the unity problem remains, we need to determine why these two chosen structures (types, systems, minds, or modes) each form a unity with individual properties, or what the mechanisms that explain this unity are.

The current manuscript attempts to advance in the unity problem by showing how T1 features align with predictive processing and how T2 features align with symbolic processing. Sloman (1996) has done a similar job with the theories of the 90s. However, his project was not developed along the years. Since there have been a multitude of related dual process theories (see Evans, 2008) with different features proposed to explain different areas of cognition, Evans and Stanovich (2013) had to review what the main features for the case of reasoning, judgment and decision making are. Further development in terms of fast or slow responses have also been proposed by Kahneman (2011) and De Neys (2017). Previous attempts at approaching the unity problem like Epstein et al. (1996) and Sloman’s (1996), therefore, refer to different theories altogether. The view that there is an “associative” system 1 and a “rule-based” system 2 is somewhat out of line with the developments both of current DPT and current cognitive architectures, like predictive processing. Moreover, there are newly discovered characteristics specific to predictive processing that explain T1 features more than an associative account does. Hopefully these characteristics will be made clear along the argument.

Perhaps a weak spot of the current proposal is that for it to stand, two other hypotheses need to be true:

(1) Predictive processing is aligned with embodied cognition.
(2) Current formulations of DPT adequately explain reasoning, judgment, and decision-making.

Although I will attempt to explain and defend these two hypotheses along the manuscript, I cannot make a full case for each of them here. Hypothesis 1 is defended mainly by Clark (2013a,^2015^, ^2016^) and although hypothesis 2 stems from the reasoning, judgment and decision-making literature starting from the 60s, the current formulation of the theory is what needs to hold (Schneider and Chein, 2003; Kahneman, 2011; Evans and Stanovich, 2013), with some emphasis given to speed, explicitness and implicitness, autonomy, and working memory.

The manuscript is organized to reflect how these features of DPT can be best captured by each of the two considered cognitive architectures. But before getting into the argument, I start by summarizing how Clark (2016) has argued that predictive processing is embodied. Then, I explain which features of DPT will be considered. I then lay out the general hypothesis for how predictive processing and symbolic accounts of cognition could go together to explain human reasoning. Finally, I go on to argue in a few sections that this hypothesis is plausible by showing how it explains the different features of T1 and T2 processing accordingly.

## How Predictive Processing Is Aligned With Embodied Cognition

Although any cognitive proposal speaking of representations and brain circuits were previously considered to be distanced from embodied cognition, Andy Clark (2013a,^2015^, ^2016^) has recently published extensively on how predictive processing can go along with or even enrich embodied, situated, and extended accounts. Philosophers have displayed worries that Andy Clark, by adopting predictive processing, had moved to a different camp.

Predictive processing suggests the brain is in a active cycle of predicting what will perturb it in a proximal and distal future. Instead of being understood as reading input from the world, the predictive brain uses statistics to anticipate input before they arrive. These predictions are based on expectations (or a statistical generative model) which foresees the most likely outcome of stimuli.

These models suggest the brain is formed by a hierarchy of processing (comprising higher and lower levels) where multiple layers of neurons are organized to compose a network with two major streams of information flow. On the top-down flow, each higher layer attempts to predict the workings of the one underneath it. The bottom-up flow conveys error correction on previously attempted predictions to each layer above. If predictions of a given event are on track, lower sensory stimulation is attenuated. On the other hand, if predictions are misleading, sensory stimulation flags the difference between what was predicted and what was perceived so that the system tries to overcome such gap. This, prediction error minimization, Clark (2013a) claims, is the brain’s major goal.

Clark (2013a) notes an interesting shift the predictive processing approach suggests. It proposes that the forward flow consists not so much of all the features that were detected to be passed onward to higher layers but only the error necessary to correct and update models. Instead of conveying all information from the environment, rather, it provides a natural funnel which guarantees processing economy by focusing on newsworthy information in the form of error correction. Predictions flow downward at each layer and error correction escalates upward showing faults to be corrected for future models. Thus, lower layers bring novelty since they detect the most recent error correction to propagate upward, but the higher layers have error correction coming from various other strands of the network. That is, the higher layers have models corrected from various sources while the lower layers will have tokens of newest corrections to be made, that is why at any given time there is not one generative model but various co-evolving models and also why there is a bidirectional flow of information.

Prediction error is also related to the concept of surprisal. Predictions are based on models which are a form of subpersonal expectation. When these expectations are not met, prediction error flags them with surprisal. In order to predict, the brain is always attempting to find a match from higher expectations to the next information reported from the bottom. Surprisal occurs, therefore, when there is a mismatch between expectation and the information conveyed by error signaling. The goal of the system at every second is to minimize surprisal. To reach such goal it must constantly update its models in order to correspond to novelty. Having tuned predictions enables the system to keep surprisal at the lowest level possible.

One of the issues in considering predictive processing as an embodied framework is its intensive use of representations to explain cognition. Embodied and situated cognition had as one of its central tenets that cognitive science had lost itself in the use of cognitive representations, and that the world itself could serve as its best model.^1^ When Clark (2013a) then claims that for every aspect of cognition the brain keeps statistical models of reality at first this seems like a huge departure from situated approaches. But it is not so. First, these representations are nothing like symbolic stand-ins, they are not mirrors of reality, and there is not an inner token for each outer stimuli. In predictive processing, these statistical models keep information only of organism-relevant stimuli and events, generating predictions that enable the organism to select affordances (see Gibson, 1979). The word ‘model’ might also sound misleading here. A model airplane is a replica of a real airplane. However, a statistic model bares a sort of morphism relation to some content, but it does not replicate the content. Further, Clark argues these statistical models do not address an organism neutral world nor even all the aspects that could be relevant to the organism. Unlike classical models, these representations are not stored in blocks and do not cause overload resulting in computational explosion, rather, Clark argues these models have been mathematically studied and found to be extremely feasible and have been applied cheaply to computer simulations. Also, Clark notices there is a sense in which the world can be its best model even if models are guiding perception, no contradiction included. The reason is that these models are not replacements for the world, instead they enable the agent to use the best of what is available in the world. If you follow this trend, the world is not its best model in a literal sense, because (unless you have very specific sensors like insects) the world actually has a majority of irrelevant information for a given agent, just think of a loud, noisy city. There is a sense in which the world is bombarding us with bad information and noise. The true sense of the expression “the world is its best model” is actually preserved by Clark. That is, that our prediction mechanisms should be at each millisecond corrected by errors in the environment, thus the environment really is what shapes us, but we need to let the right information shape us, not any irrelevant information from the environment. Generative models actually permit us to be tuned to the human-relevant environment.

Another issue is that of the implied metaphysics. If our systems only get information (error) relative to predictions, does that imply indirect perception? Clark’s (2016) answer is yes and no, or “non-indirect perception.” The worry of critics of indirect perception is that we might be locked from the true world itself. The point, once again, is both that we need the mechanisms to engage in the relevant world and that the world itself, as in free from agent intentional perception, is senseless. When we go to the stadium, predictive processing is what enables us to see a soccer game instead of physical objects colliding. Therefore, Clark argues perception cannot be direct since it is mediated by expectations, but no further worry needs to be pursued about “losing the world.” This is because predictions allow us to see the part of the world that is relevant to humans, without these models, if we could perceive at all, a random part of a scene would be as relevant as a face.

Finally, there is the embodied coupling of perception and action. This is achieved in predictive processing because actions are a consequence of external and proprioceptive perception and because action reduces prediction error by directing what sort of stimuli perturbs the sensory system. Therefore, to solve a jigsaw puzzle we need to actively engage the objects with our hands, rotating, moving and organizing them, and in every such attempt, action is framing the sort of stimuli that perception will receive, choosing what “shots” of the world are taken. This interplay between action, body and world is what solves a tough jigsaw puzzle, one cannot succeed just by staring at it and thinking. Clark (2016) shows how embodied proposals of the mind can assume diverse shapes. His version might not be very representative of the movement, however, if embodied proposals of the mind are to be relevant to cognitive science, then these must adopt or develop models of cognition like Clark does with predictive processing.

## Features of Dual Process Theory

Dual process theories come in various shapes. If we simply put all dual process theories that have been proposed together we arrive at a multi-theoretical cluster of attributes for each type of processing (Evans, 2008), thus the correlational features for T1 processes would be: unconscious, implicit, automatic, low effort, rapid, high capacity, default, holistic, perceptual, evolutionary old, follows evolutionary rationality, shared with animals, non-verbal, modular, associative, domain-specific, contextualized, pragmatic, parallel, stereotypical, independent of general intelligence, independent of working memory. In this multi-theoretical cluster version, the correlational features for T2 processes would be: conscious, explicit, controlled, high effort, slow, low capacity, inhibitory, analytic, reflective, evolutionarily recent, follows individual rationality, uniquely human, linked to language, fluid intelligence, rule based, domain general, abstract, logical, sequential, egalitarian, heritable, linked to general intelligence, limited by working memory capacity. Evans (2008) noted that positing all these features as defining characteristics of these types of processing is troublesome, because these characteristics will not always stand.

It is quite improbable that such a strong co-occurring requirement meets reality. Because even if, say, only six dichotomies are advanced, there are still 64 possible combinations of these features that need always co-occur. If DPT were proposing such an alignment assumption for all these features (see Stanovich and Toplak, 2012) then only one of these possible 64 combinations of features would be enough to falsify the theory. Suppose these dichotomies were: conscious/unconscious, explicit/implicit, controlled/automatic, serial/parallel, slow/fast, resource dependent/resource free. Each process that lacked one element of these aligned features would serve as evidence to falsify DPT. For example, a process that was conscious, explicit, controlled but parallel would be evidence for falsification, even considering that most features of such process were rather aligned than unaligned.

Critics have mentioned how DPT features are not well defined (Keren, 2013). However, one can reformulate this theory to account for new evidence. We just have to be aware that if this happens repeatedly, we should start losing our interest in DPT (see Lakatos et al., 1979). The correct way to go about this is to try to consider which would be the crucial features of dual process theories of reasoning such as Schneider and Chein (2003), Kahneman (2011), and Evans and Stanovich (2013) have attempted. This should be at least a combination of features which various theorists of this research field or similar research fields could agree on. By assuming the alignment assumption at least for defining features, the theory gains in predictive power and rigor. Therefore, the more defining features one assumes, the stronger are the empirical consequences; it will predict more but also be more easily false. At least for defining features, predetermined scientific predictions must be possible, or else these features are not truly defining.

Based on the weight placed on these features in the works Schneider and Chein (2003), Kahneman (2011), and Evans and Stanovich (2013) we will focus on five main dual process distinctions: working memory use, explicit and implicit representations, automaticity, and speed.

## Hypothesis

To solve the unity problem, I propose a hypothesis to combine embodied predictive processing with symbolic classic approaches. The hypothesis, simplified, states that T1 features form a unity because they rely on embodied predictive processing whereas T2 processes form a unity because they are accomplished by symbolic classical cognition.

Daniel Kahneman (2002, p. 450) wrote that “From its earliest days, the research that Tversky and I conducted was guided by the idea that intuitive judgments occupy a position […] between the automatic operations of perception and the deliberate operations of reasoning.” Kahneman and Frederick (2002, p. 50) claimed that intuitive thinking is “perception-like” and that “intuitive prediction is an operation of System 1.” Further, that “The boundary between perception and judgment is fuzzy and permeable: the perception of a stranger as menacing is inseparable from a prediction of future harm.” Kahneman et al. (1982) have been speaking of “intuitive predictions” for a long time. What I hold is the link between perception and intuition obtains because T1 judgments are embodied predictions. These authors have been noticing that intuition is somewhat like perception and have used the term prediction as what intuition does, but they have not argued for a framework for T1 processes.

Perception clearly has input functions, but what is interesting for DPT of reasoning and decision making is that T1 processes have an output function, in the sense that they generate answers to problems. The predictive processing approach gives a clear output form to perception, by emphasizing its generative character. Thus, a strong claim I want to hold is that T1 processing answers (or output functions) are predictions.

The pivotal role of expectations for determining T1 predictions have gone mostly unnoticed even though task construal in the reasoning and judgment paradigm has been mostly a task of manipulating subject’s expectations. The argument for how this occurs in reasoning is that T1 processes take information over prior occurrences and over the current set of states (likelihood) and yields a fast prediction (posterior). If the time constraint is rigid, these predictions will generate actions (inner mental responses or, if too rigid, movements). If the system has time, then these predictions will be available for T2 evaluation. Thus, T2 processes receive T1 predictions as input to analyze and possibly override. That is why manipulating subject’s expectations in a task causes their T1 answers to vary accordingly and requires T2 effort to override them.

According to the current hypothesis, T1 processes deal with content encoded in the form of probability density functions, which means there is no symbol and no definite content, but values, means and standard deviation influenced by previous movements and previous world contingencies. Manipulating prior information biases the distribution into one or another direction, closer to or further from a certain value. These functions are not stored in a memory bank but distributed from the responsible brain regions over to external organs and body parts through neural connections. The values in the distribution do not represent objects directly and discretely, they refer to distinct aspects of the input when perceptual systems are dealing with such objects. This is in line with T1 processes being easily biased when working with references to similar properties, like similar numbers, objects, rhymes or pet names; very often the incorrect value is picked from a distribution. This is also in line with claims of embodied proposals that the world is not represented in symbols.

Finally, T1 processes are subpersonal (see Frankish, 2004, 2009) and their predictions are made by the same systems which process perception. A clear example is that a judgment (a prediction) about facial expressions is related to the FFA (see Egner’s et al., 2010). The idea is that perception is not passive but already comes with predictions, and when in problem solving, such prediction is precisely the T1 answer. I do not want to claim that T1 processes are purely perceptual (if in contrast to cognitive), only that such predictions stem from perceptual processes. Kahneman’s (2011) example of judgments of angry facial expressions shows how this is expected of DPT. Kahneman (2002) and Kahneman and Frederick (2002) have also argued that the list of features of T1 processing is shared with perception mechanisms. What I propose to do is examine central T1 features to show that it is shared because both (or at least part of) perception and T1 processes work in the manner described by predictive processing, which is also in-line with the claims of embodied cognition that there is no sharp link between perception and reasoning.

It is interesting to note that Clark’s (2016, p. 257) embodied version of predictive processing is described accordingly: “Fast, automatic, over-learnt behaviors are especially good candidates for control by models taking a more heuristic form. The role of context-reflecting precision assignments is then to select and enable the low-cost procedural model that has proven able to support the target behavior. Such low-cost models […] will in many cases rely upon the self-structuring of our own information flows, exploiting patterns of circular causal commerce (between perceptual inputs and motor actions) to deliver task-relevant information ‘just in time’ for use.”

Another way to put it, which fits neatly with the framework developed here is: “we need only note that very low-precision prediction errors will have little or no influence upon ongoing processing and will fail to recruit or nuance higher level representations.” (Clark, 2016, p. 148) That is, if the task is overlearned and errors are weighted as low, systems will act without further recruiting. This can be understood as a hypothesis for automaticity, which has been used so much in psychology but without an explanation for why it differed from controlled processing.

The general idea I want to hold for T2 processing is that it works like a classical machine for reasoning, such as the General Problem Solver (GPS, Newell and Simon, 1963). The GPS was one of the first attempts to mimic human reasoning. Its purpose was to respond to logical problems like humans would. Of course, human thought is different in various ways from those first machines; but T2 processes are somewhat alike. However, this classical machine only makes sense in the brain if it exists in the wider setup of a predictive processing network generating T1 responses.^2^ Thus, like Newell’s (1980) physical symbol system, when facing a reasoning problem, T2 processing opens a problem space containing an expression that designates the initial problem (how it was digitized or interpreted) and an expression that designates a solution, which was produced by a probabilistic prediction (T1 processing). Having the initial expression and the predicted expression in the problem space, T2 processing then uses its move generators to attempt to reduce differences between them and sometimes finds different solutions in such path or illuminates something that previously had not come about. Move generators (or operators in the GPS) are mechanisms that apply rules, which might be fed from different sources, such as logic, mathematics or philosophy (say Occam’s razor). These generators are likely to be flexible, in that they can change depending on the problem. Thus, although the basic structure is that of a logical machine that works on symbolic expressions it could be set up to apply paraconsistent rules, for instance. This is possible because although it does not work with contradictory expressions it could work with expressions that designate contradictory expressions. Therefore, it is free to work out any sort of principle to solve tasks, exhibiting the property known as universality in computation.

I want to make it clear that I am taking “classical architecture” and “predictive processing” both as whole packages. Computations have universal features, classical architectures could work with representations of probabilities and predictive processing could be realized by a serial machine. But this is out of their standards. To claim that I am taking the whole package means that I am taking features of classical architecture and predictive processing that usually come together in all levels. Therefore, I am speaking of a classical architecture in the form of a serial physical symbol system performing heuristic search such as a GPS (Newell and Simon, 1963, 1976; Newell, 1980) which are responsible for T2 processes and embodied prediction as a hypothesis about how networks in the brain form a system with the body that encodes probabilistic representations of stimuli which are used to infer properties of objects in the world, and act upon them being responsible for T1 processing (Clark, 2013a,^2016^; Hohwy, 2013).

Some caveats are in order. We should not want to suppose that there are two processes for the mind as whole, since that would be too strong of a hypothesis and evidence from any cognitive function would serve to falsify it. Therefore, it is important to restrict this hypothesis first to the scope of reasoning, judgment and decision making. Also, a huge list of features have been ascribed to DPT (see Evans, 2008) and it might be the case that some do not follow the current hypothesis. Although I have not identified such features that would not work at all with such hypothesis, Evans (2008) argues that this group of features cannot work coherently together, so some must be off track. Decoupling is an important feature which was not mentioned here, but that is because it requires extensive work, and the manuscript is limited by space. Interestingly, if this hypothesis stands to empirical tests and there is further reason to believe it, then it could even help expose those features from Evans (2008) which were off track.

This is the general hypothesis. None of what is claimed so far is novel in itself, just in the interpretation of how these claims could work together. To show that this interpretation is likely true, I will proceed by showing how central T1 features are best captured by predictive processing and how central T2 features are best captured by classical architectures.

## Implicit and Explicit Features

Although the “implicit” and “explicit” distinction is vastly used in the literature in the sense of access, this is also the use of “consciousness.” When it comes to the implicit and explicit distinction what is unique and coherent (even with the word) is the representational format (see Bellini-Leite, 2021). If we want a difference between the explicit and implicit features in DPT we need to have different representational formats for each type of process.

Predictive processing has a unique representation format, content is encoded in probability density functions. These functions these functions do not disambiguate items discretely, rather, they gather multiple occurrences of events and possibilities from models ranging from various areas of the cortex, body and world contingencies to generate probability. This is most likely the (usually unexplained) meaning of an implicit format in cognitive psychology, one that encodes probability of previous occurrences of movements and world contingencies and not representations by means of symbols. This implicit format is not the type of format T2 reasoning can work with, T2 processes need symbolic, unit-like objects to reason over, and that is the meaning of an explicit representational format: disambiguated stand-ins for a unified object.

A representation is explicit when it has a graspable representational format. By this I mean that subjects seem to grasp such content with ease and they verbally report having done so. This contrasts with fuzzy content which one does not know how to speak of or even think clearly about. It seems we can be conscious both of fuzzy and disambiguated content.

Classical architectures can have more fixed access to the content it deals with than predictive processing networks because of differences in symbolic representations and probability density distributions. Probability density distributions are responsible for much of what gives predictive processing its explanatory success. Representing information in such fashion allows for statistical processing of previous input and for generative guesses for future outcomes involving diverse elements distributed between the cortex and the world. There is a problem with this representation, however, which is keeping a probabilistic take on states of objects, since it includes too much. Having this probabilistic state usually allows embodied agents to act more rapidly, but there are times when we need precise, definite, properly discrete information about an object. In such times, only one answer is valued and related ones should not interfere. To account for this, Clark (2016) speaks of single peak probability distribution functions, representations where each distribution must have a single best explanation. Thus, instead of having various related peaks indicating possible outcomes of movements and world contingencies, only one is enforced. “One fundamental reason that our brains appear only to entertain unimodal (single peak) posterior beliefs may thus be that—at the end of the day—these beliefs are in the game of informing action and behavior, and we can only do one thing at one time.” (Clark, 2016, p. 188).

Now, what happens when you have a single peak probability density function is that it acts like a discrete symbolic representation. That is, all other possible states are denied in favor of a single active state. When this is the case, advantages of embodied prediction of using statistical encoding and generative models over the multitude of possible body-world relations are lost and some other form of computing needs to take place. When using single-peak probability density functions you lose the effects of having various related instances as possible outcomes to gain feasibility, you lose effective predictive processing.

Clark (2016) admits that sometimes values in a density function need to be reduced to only one. However, what goes by unnoticed is that this is precisely the effect of turning it into a symbolic representation. This eliminates uncertainty, and possibly is related to subjects being able to grasp the content. You can grasp something that is clearly defined but you cannot easily grasp the meaning of something like values in a probability density function. They are fuzzy because they cannot be simply well defined. It is precisely their fuzziness that allows for context-sensitivity and fluid embodied cognition.

The reason classic symbols are graspable seems to be because working memory can store them and use them in symbolic manipulation. Working memory cannot store all values of a probability density function or manipulate the dynamic workings of a complex relation between movements and world contingencies. But when this whole dynamic is referenced by a single symbol, this symbol can then be treated as a constituent in an expression. When that occurs, the classical architecture can work with compositionality (see Fodor and Pylyshyn, 1988).

As Fodor and Pylyshyn (1988) have explained,^3^ the point for compositionality in making content graspable is that manipulations of these expressions can then be easily tracked. Rules and semantic content become related to the inner structure of the computation. Then, when taking some content as a symbolic object, it becomes identifiable in multiple expressions preserving its identity. In contrast, values in a density function might lose their identity, in fact, we should want that to happen if context is to shape their identity.

Even the steps in processing can become symbols themselves by being stored as expressions to be used in metacognition. Therefore, when we are reasoning in a syllogism, we can keep premises in working memory and also the steps used to extract one from the other. Of course, these are fleeting, but also, the way to make them less fleeting is by reducing uncertainty and naming a step or a premise by a letter or a simple symbol, say MP. So it seems plausible that representations in classical architectures should make both content and steps of processing more graspable because of ease in determining their identity, reducing uncertainty. Therefore, if the current hypothesis holds, we should want to speak of explicit representations as symbolic and implicit ones as distributed, probabilistic, and multi-valued.

## Automaticity Versus Working Memory

Automaticity concerns overlearned skills, and overlearned skills here can be understood as skills over tasks that became predictable. Let us use the classic example of learning how to drive a non-automatic car to see how predictive processing relates to automaticity. When we first sit behind the driver’s wheel, even if we have knowledge on what must be done, our systems cannot coordinate all such knowledge in order to be useful (and safe). When we train ourselves the correct order of using gears, wheel turning and pedals, we are tuning our predictive processing systems to the usual occurrences of car handling. Of course, before driving, our systems cannot have useful priors on the matter. By letting our system engage with the stimuli necessary for driving we tune it to that particular context, that is, we learn embodied/predictive routines. For instance, when in cliffs, our systems need to predict the exact moment to press the clutch at the correct strength to manage the cliff. But not only this, our systems need to predict more precisely when another car is stopping in front of us. They need to predict the order of gears and when they will be necessary, also when the car is being misused through auditory clues.

Various cues are used to predict near-future occurrences. The system needs to know, for various states, that if it is in a given state, another given state is the most probable to follow. Once the system learns various important cues that lead to efficient predictions, it can handle most driving abilities automatically. Thus, an experienced driver will incur in far less surprisal instances than a novice driver. In fact, the higher surprisals which will come by are in the form of unpredictable changes in the environment, such as an animal crossing the road. In contrast, the surprisal which will mostly concern the novice is in terms of actions to handle the machine, so an animal can go by unnoticed. If our systems have no useful priors for driving, they need to rely on effortful controlled skills to train predictions systems, but these effortful controlled skills cannot be predictive processing skills themselves.

Unlike driving, daydreaming seems to be turning attention and effort to oneself and forgetting the world for a while. What seems to happen to attention and working memory in predictable situations is that it turns inward, it starts to generate novelty or monitor inner performance. This is observable in habituation, a phenomenon much known by psychologists where exposure to repeated stimuli decreases attention paid to it. Working memory is an online and ever-ready mechanism for dealing with further uncertainties and unpredictable information. It seems to be that the more predictable a given state is, the less working memory resources systems will consume in processing it. Working memory is needed when predictive processing fails.

The literature in predictive processing does not necessarily shun working memory, but just to illustrate how important this concept is to such framework, it is interesting to see how it is mentioned only once in Clark’s (2016) book and absent from Hohwy’s (2013) book and other work in predictive processing. Working memory is mentioned 119 times in Frankish and Evans’ (2009) review of DPT. In other words, it is probably not a very central tenet of predictive processing. And there is every reason for working memory not to be a relevant tenet of predictive processing. This is precisely because stronger load on working memory concerns cases where the information that needs processing is unpredictable, or is not well accommodated by any statistical judgment, in fact, if the general prediction by statistics schema fails deeply to account for some relevant data, then it seems plausible that another type of processing should be applied. When predictions are working, then, working memory is mostly dispensable.

Working memory is not a feature of how predictive networks work. In contrast, a working memory is a necessary component of a classical architecture, both structurally and functionally. Thus, I argue that it is unlikely that predictive processing can do away completely with models of classical processing as proponents usually hold.

In a Von Neumann (1945) architecture there is a primary storage for holding what to do and what is done, which is basic for the functioning of the machine. More importantly, in a physical symbol system, the model proposed by Newell (1980) for classical cognitive science, a similar component that stores operators and expressions which are being used at a given moment is necessary. In Newell’s (1980, p. 159) words “This organization implies a requirement for working memory in the control to hold the symbols for the operator and data as they are selected and brought together.” and “[…] working memory is an invariant feature of symbol systems.”

A working memory in cognitive psychology is usually taken to be a system with executive functions and not only a storage. As Baddeley (1992, p. 557) explains “Although concurrent storage and processing may be one aspect of working memory, it is almost certainly not the only feature.” In fact, it is such executive functions which pushed the need for the concept of a working memory instead of just a short-term storage. Baddeley (1992, p. 556) explains that “This definition has evolved from the concept of unitary short-term memory system. Working memory has been found to require the simultaneous storage and processing of information.” Instead of being just a short-term storage, the model also includes “an attentional controller and the central executive, supplemented by two subsidiary slave systems” (Baddeley, 1992, p. 556). These slave systems are storages for different types of content, such as phonological or visual. More important for present purposes are the “attentional controller” and “the central executive.”

It seems these claims on the processing abilities of working memory are not as clear as what has been said of its storage function. For instance, Baddeley (1992) claimed that the attentional controller was an additional component, but he also claims “the central executive […] is assumed to be an attentional-controlling system.” We understand executive functions are equivalent to the application of operators in Newell’s (1980) architecture or to the functioning of a processing unit of a Von Neumann architecture which carries out logical or arithmetic procedures. As for the attentional controller, it is not directly related to attention as in the psychological concept, but to “attention” as in a Turing machine which can only focus on certain elements each moment. This function would also be something like the control unit of the Von Neumann architecture which mediates the flow of processing by providing timing and control signals. With the argument that T2 processing depends on working memory, what is meant is that that a temporary storage is needed but also other mechanisms which mediate symbol processing, or that something like the physical symbol architecture of Newell (1980). Certain operators must be applied to elements of this storage and there must be a control of which expressions are being used at a given moment.

There are two choices here, one is to say that the concept of the working memory refers to Newell’s (1980) physical symbol architecture as a whole, or that it is the storage component of such architecture. Since the literature (Baddeley, 1992) sustains the importance of executive functions which differentiates working memory from the concept of short-term memory, the first choice seems more plausible: that working memory is not only a memory, but a system which has very similar (if not the same) properties to that of Newell’s (1980).

Newell’s (1980) architecture maintains properties of a Von Neumann architecture which maintains (or instantiates) properties of Turing Machines. By transitivity (and if the hypothesis is on track) there should also be some similarity between working memory and Turing machines. First, it is enlightening to notice that Turing started to think about his machine by trying to mimic what he was doing in his own abstract thought, such as the processes he was executing when doing mathematics. Thus, since we must process in working memory what we are thinking consciously and with effort, which clearly was the type of thought he had to engage in for his work, what he probably was doing then was an inspection of the functioning of his own working memory. If this supposition is the case, it would also be no surprise to find similarities of working memory and a Turing machine.

Consider this part of Turing’s (1936, p. 250) intuitive argument: “The behavior of the computer at any moment is determined by the symbols which he is observing, and his ‘state of mind’ at that moment. We may suppose that there is a bound B to the number of symbols or squares which the computer can observe at one moment. If he wishes to observe more, he must use successive observations. We will also suppose that the number of states of mind which need be taken into account is finite. The reasons for this are of the same character as those which restrict the number of symbols. If we admitted an infinity of states of mind, some of them will be ‘arbitrarily close’ and will be confused. Again, the restriction is not one which seriously affects computation, since the use of more complicated states of mind can be avoided by writing more symbols on the tape.”

This description is like that of working memory in various ways. We can see that clearly by switching the term “computer” with “working memory” in this quotation. By doing so, every claim continues to be true. If fact, he could just as equally be describing working memory:

(1) The behavior of working memory at any moment is determined by the symbols which he is observing, and his “state of mind” at that moment. (2) We may suppose that there is a bound B to the number of symbols or squares which working memory can observe at one moment. (3) If working memory wishes to observe more, it must use successive observations. (4) We will also suppose that the number of states of mind which need be taken into account is finite. (5) More complicated states of mind can be avoided by writing more symbols on the storage components of working memory.

This paraphrasing in Turing’s words would not work were we to use “predictive processing” or “T1 processes.” The statements would then be false. It seems like Newell’s (1980) architecture is adequate in many ways to serve as a model of working memory whereas predictive processing is not.

T2 processes are those that load heavily on working memory, and thus, are likely executed by a system like Newell’s architecture. On the other hand, of course working memory processes could only be restating what T1 processes have already arrived at. This possibility is show, for instance, by the computerized version of the Wason selection task (Evans, 1996). Also, it is allowed by definition that T1 processes might load weakly in working memory. A possible option is that for us to consider a token process as T2, conclusions to such problem must be reached only after the use of such distinct computational methods of Newell’s architecture. That is, something must be found in heuristic search (see Newell and Simon, 1976) which was not found in predictive processing in order for a process to be considered T2.

A stronger hypothesis is that human working memory is literally a classical architecture simulated by the brain, or a component of such, and also that its executive functions are literally the application of operators as in Newell’s symbol systems. This would be a problem if the whole mind was said to work in this fashion. But in this case it is only T2 processes that are realized by such architecture, which are a very limited class of mental functions. A weaker hypothesis would be that T2 processes have similar features to that of classical architectures, but there is no metaphysical commitment implied. Either one does the job of solving the unity problem for the working memory feature.

## Speed

Time is valuable for the effectiveness of T2 processes. As we know, the first computers ever invented were much slower than the ones we have today. Thus, having the best hardware for processing in a given way is tantamount to fast processing. In contrast, the brain and the body are a network of cells, so simulating a classical architecture is not what is natural of it.

That we organize our goals explicitly and that we investigate possibilities better than other animals seems to be true. It also seems to be true generally that we are better at T2 processing than other animals are. For instance, no other animal knows what mathematics is, and are not able to explore consequences of axioms (although, of course, they can know about quantities). So it seems to be true that T2 processes are an unnatural function of the mammal brain. If we follow the hypothesis that T2 processing is the result of operations of simulated classical architecture in the brain, then it would make sense to assume that such simulated architecture does not have the appropriate hardware conditions to perform with the speed of computers built just for such functions.

Following the hypothesis, we should want to claim that classical architectures are slower than predictive processing architectures. We do not have computers with hardware in the forms of networks, much less ones that compute probabilistically in such hardware. We only have simulations. Anyhow, we do have reason to believe that networks are faster. As Fodor and Pylyshyn (1988, p. 35) comment: “in the time it takes people to carry out many of the tasks at which they are fluent (like recognizing a word or a picture, either of which may require considerably less than a second) a serial neurally instantiated program would only be able to carry out about 100 instructions many thousands—or even millions—of instructions in present-day computers (if they can be done at all).”

Of course, by defending classical architectures, Fodor and Pylyshyn (1988, p. 39) go on to argue that these are issues of the implementation level. In fact, that any speed issue should be so. “The moral is that the absolute speed of a process is a property par excellence of its implementation.” If this is the case, then apparently, we have two reasons to think that T2 processes in the current developing framework would be slower. First because network processing will tend to be faster in comparison and second because, as physiology teaches us, the brain does not have the appropriate hardware for the implementation of a fast classical architecture. However, although Fodor and Pylyshyn (1988) were correct that implementation relates to speed, they were wrong in claiming that speed is determined solely by implementation. Using explicit steps over discrete symbols implies certainty over speed. Even in speech we can note how we avoid communicating every explicit step of our thoughts but rather leave open implicit assumptions that are never spoken, in order to maximize speed.

In contrast to favoring certainty over speed, to defend predictive processing’s speed, Clark (2016, p. 250) claims “Cheap, fast, world-exploiting action, rather than the pursuit of truth, optimality, or deductive inference, is now the key organizing principle.” Surely, a cognitive architecture that attempts to predict incoming information surely must have a recipe for being faster than others. A predictive processing architecture can act faster because any cue captured from the world is readily met with predictions (even if bets) concerning a lot more than the cue itself shows. The predictive processor is always taking certain bets about what the current state of the world implies, losing accuracy in compensation for speed. So it fits nicely with the idea that T1 processing needs to abandon certainty and accuracy for speed, an idea previously developed as quick and dirty heuristics (see Gigerenzer, 1996). Predictions are also quick and dirty and perhaps in a way that makes these properties even more ubiquitous since it spans even perceptual details and not only judgments. Thus, when watching a white scene in a movie, there might be guesses that there are no black and brown pixels in some areas of the screen, even if there are. The quick and dirty guessing thus extends far beyond what traditional frugality theorists (i.e., Gigerenzer, 1996) had been considering.

Another property that allows for fast processing is predictive coding (Rao and Ballard, 1999). By predictive coding we mean specifically the property of these system to consider, from the world, only stimuli which result in greater prediction error. Thus, some stimuli are considered in real-time perception already as irrelevant for the adaptive use of the organism. Precision weighing (see Clark, 2013b) quickly determines the size or effect of the prediction error determining if it is eliminated or if it needs to further propagate to other areas. Focusing on prediction-relevant stimuli only permits the agent to quickly decide courses of action and to select amongst possible affordances (see Gibson, 1979). T1 processing can thus be understood as quick predictions emerging from the system’s first considerations of these errors.

As Clark explains embodied prediction, the agent is always tuned to environmental cues which can quickly help the system decide between affordances. The predictive architecture provides means for quicker selection, “allowing time-pressed animals to partially ‘pre-compute’ multiple possible actions, any one of which can then be selected and deployed at short notice and with minimal further processing.” (Clark, 2016, p. 180). In the cases studied by DPT, mostly of people taking reasoning and decision-making tests, this quickness of action comes in the form not of body movements but of simplistic hypothesis quickly springing to mind. Such hypothesis come to mind quickly because of the probabilistic relations they bear with the input. So we can even start to ponder about the basis of accessibility, which worries Kahneman (2002, p. 456) “much is known about the determinants of accessibility, but there is no general theoretical account of accessibility and no prospect of one emerging soon.” Accessible content could be understood as the higher values in probability density distributions of a generative model related to the range of possible responses to a given task. The more given values have been used to reduce prediction error in the (evolutionary and developmental) past the more the content will be accessible.

## Conclusion

I have argued that many T1 core features are necessary features of a predictive processing architecture, whereas classical architectures cannot be done away with and its mechanisms are functionally presupposed in T2 processes. Taken together, various reasons were given for this hypothesis to hold in relation to representational format, automaticity, working memory and speed. This endeavor is meant to solve the unity problem as posed by Samuels (2009). It is of central importance to understand why there are two property clusters of processing features for reasoning and decision making and DPT needs further theoretical development to defend it from recent attacks (see Osman, 2004, 2013; Keren and Schul, 2009; Kruglanski and Gigerenzer, 2011; Keren, 2013; Kruglanski, 2013; Melnikoff and Bargh, 2018).

For the future, we need other associated projects to test this hypothesis. From psychology we need to see if evidence does hold for T1 answers as stemming from predictive processing and T2 as following a classical architecture. From artificial intelligence we need to see that such a hybrid is useful and feasible. Neuroscience should be able to detect different types of related mechanisms in classical reasoning, judgment and decision-making tasks, not too much in brain region but most likely in action potentials. Altogether, this is a hypothesis that needs to be investigated, rather than taken as correct. Although the arguments hold, only empirical evidence will show if it is true or false.

## Data Availability Statement

The original contributions presented in the study are included in the article/supplementary material, further inquiries can be directed to the corresponding author.

## Author Contributions

The author confirms being the sole contributor of this work, and has approved it for publication.

## Conflict of Interest

The author declares that the research was conducted in the absence of any commercial or financial relationships that could be construed as a potential conflict of interest.

## Publisher’s Note

All claims expressed in this article are solely those of the authors and do not necessarily represent those of their affiliated organizations, or those of the publisher, the editors and the reviewers. Any product that may be evaluated in this article, or claim that may be made by its manufacturer, is not guaranteed or endorsed by the publisher.

## Footnotes

1 Although it is important to note that embodied cognition comes in various forms. For instance, Glenberg (1999) prefers to speak of embodied models.

2 This is also the case for meaning in the sense of Harnad (1990). In this framework, classical symbols reference instances of predictive processing exchanges with the world.

3 Please note that there are also issues of using the classical interpretation for T1 processing which have been stressed in Bellini-Leite and Frankish (2020).
